# 吉西他滨联合奥沙利铂或顺铂一线治疗老年晚期非小细胞肺癌的随机研究

**DOI:** 10.3779/j.issn.1009-3419.2011.07.05

**Published:** 2011-07-20

**Authors:** 之曦 李, 梅 侯, 海燕 王, 泽阳 王

**Affiliations:** 610041 成都，四川大学华西医院肿瘤中心 Cancer Center, West China Hospital, Sichuan University, Chengdu 610041, China

**Keywords:** 吉西他滨, 奥沙利铂, 顺铂, 肺肿瘤, 老年, Gemcitabine, Oxaliplatin, Cisplatin, Lung neoplasms, The elderly

## Abstract

**背景与目的:**

以铂类为基础的化疗是晚期非小细胞肺癌（non-small cell lung cancer, NSCLC）的标准治疗方案。本研究旨在评价吉西他滨联合奥沙利铂和吉西他滨联合顺铂一线治疗老年晚期NSCLC的疗效及毒副反应。

**方法:**

未经过治疗的经病理学或细胞学确诊的老年晚期NSCLC患者66例随机分成GO（吉西他滨1, 000 mg/m^2^第1、8天+奥沙利铂130 mg/m^2^第1天静脉滴注，每3周重复）组33例和GP（吉西他滨1, 000 mg/m^2^第1、8天+顺铂25 mg/m^2^第1、2、3天静脉滴注，每3周重复）组33例，至少治疗2周期，评价疗效及不良反应。

**结果:**

GO组与GP组在治疗有效率（36.4% *vs* 40.6%, *P*=0.801）、中位无进展生存期（24周*vs* 18周，*P*=0.565）、中位生存期（44周*vs* 36周，*P*=0.918）等方面的差异无统计学意义，而在3级-4级贫血（0 *vs* 33.3%, *P* < 0.001）及3级-4级恶心呕吐（0 *vs* 27.3%, *P*=0.004）等方面的差异有统计学意义。

**结论:**

对于老年晚期NSCLC，一线使用吉西他滨联合奥沙利铂或顺铂两种方案疗效相当，但吉西他滨联合奥沙利铂方案治疗耐受性好，临床应用更安全。

## Abstract (en)

**Background and objective:**

Platinum-based chemotherapy is considered the standard treatment of advanced non-small cell lung cancer (NSCLC). The aim of this study is to evaluate the efficacy and safety of gemcitabine plus oxaliplatin (GO) versus gemcitabine plus cisplatin (GP) regimens as the 1^st^ line chemotherapy for elderly patients with advanced NSCLC.

**Methods:**

Sixty-six advanced NSCLC patients confrmed with pathology or cytology, who had not received treatment, were randomly divided into GO group (The patients received gemcitabine 1, 000 mg/m^2^ on day 1 and day 8 and oxaliplatin 130 mg/m^2^ on day 1 by intravenous infusion, with 21 days as one cycle) and GP group (The patients received gemcitabine 1, 000 mg/m^2^ on day 1 and day 8 and cisplatin 25 mg/m^2^ on day 1, day 2 and day 3) by intravenous infusion, with 21 days as one cycle). All patients who received 2 or more cycles could be evaluated.

**Results:**

Tere were no statistical differences between GO and GP groups in the efciency of disease (36.4% *vs* 40.6%, *P*=0.801), the median progression-free survival (24 weeks *vs* 18 weeks, *P*=0.565), the median survival time (44 weeks *vs* 36 weeks, *P*=0.918), but anemia at grade Ⅲ and Ⅳ (0 *vs* 33.3%, *P* < 0.001) and nausea/vomiting at grade Ⅲ and Ⅳ (0 *vs* 27.3%, *P*=0.004) were signifcantly different.

**Conclusion:**

The clinical efciency of GO and GP regimens as the 1^st^ line chemotherapy for advanced NSCLC in elderly patients was similar, but the toxicity of GO regimen has the tendency to be more tolerable and safer.

老年人的非小细胞肺癌（non-small cell lung cancer, NSCLC）发病率较高，随着人口预期寿命的延长，老年NSCLC患者不断增多。70%以上的患者确诊时已进展为晚期，因此化疗是晚期NSCLC治疗的主要选择。铂类药物由于毒性较强，老年患者对其耐受性差。老年晚期NSCLC患者常接受第三代细胞毒性药物单药化疗或姑息治疗。但近年来研究发现，老年晚期NSCLC患者对含铂双药化疗可以耐受，且疗效更好^[1, 2]^，但最佳的治疗方案一直在探索当中。同时，由于老年患者预期寿命短、常合并多种其它疾病等原因，老年患者的化疗更应关注治疗相关毒副反应。四川大学华西医院肿瘤中心自2007年6月-2008年12月采用吉西他滨（gemcitabine, GEM）联合奥沙利铂（oxaliplatin, L-OHP）与吉西他滨联合顺铂（cisplatin, DDP）治疗66例老年晚期NSCLC，并进行随机对照研究，现报道如下。

## 1 对象和方法

### 1.1 研究对象及纳入标准

2007年6月-2008年12月我院筛选了102例未经过治疗的老年肺癌患者，共计66例患者符合研究纳入标准：经病理学或细胞学确诊为Ⅲb期/Ⅳ期NSCLC，年龄≥70岁，有可评价的临床靶病灶，化疗前血常规、肝肾功能和心电图检查均正常，无急性感染和重要脏器功能不全表现，体能状态评分（performance score, PS）为0分-2分，预计生存期 > 12周。

### 1.2 排除标准

PS评分≥3分；出现脑转移；重要脏器功能不全；合并其它恶性肿瘤；靶病灶区域曾接受过抗肿瘤治疗。

### 1.3 随机分组

66例Ⅲb/Ⅳ期NSCLC患者经SPSS 13.0统计软件随机分成接受吉西他滨联合奥沙利铂化疗的GO组（33例）和接受吉西他滨联合顺铂化疗的GP组（33例）。其中GO组Ⅳ期患者18人，有肝转移患者3人；GP组Ⅳ期患者22人，有肝转移患者4人。两组患者基线特点（男女比例、中位年龄、PS评分、疾病分期情况、组织学分型、吸烟等）无明显差异。本研究纳入患者的临床资料见表 1。

**1:** 本研究纳入患者的临床资料（*n*=66） Clinical characteristics of patients included in this study (*n*=66)

Characteristic	GO group (*n* =33)	GP group (*n* =33)	*n*	*P*
Gender				0.415
Male	22	25	47	
Female	11	8	19	
Age (year)				0.806
Median	73	74		
Range	70-82	70-79		
Performance score				0.547
0-1	25	27	52	
2	8	6	14	
Histology				0.899
Adenocarcinoma	15	13	28	
Squamous cell carcinoma	17	19	36	
Others	1	1	2	
TNM stage				0.314
Ⅲb	15	11	26		
Ⅳ	18	22	40	
Cigarette smoking				> 0.999
Smoker	18	17	35	
Non-smoker	15	16	31	
GO group: The patients received gemcitabine 1, 000 mg/m^2^ on day 1 and day 8 and oxaliplatin 130 mg/m^2^ on day 1 by intravenous infusion, with 21 days as one cycle; GP group: The patients received gemcitabine 1, 000 mg/m^2^ on day 1 and day 8 and cisplatin 25 mg/m^2^ on day 1, day 2 and day 3 by intravenous infusion, with 21 days as one cycle.				

### 1.4 治疗方案

全部患者接受联合化疗。GO组接受吉西他滨1, 000 mg/m^2^第1、8天静脉滴注+奥沙利铂130 mg/m^2^第1天静脉滴注，每21天重复；GP组接受吉西他滨1, 000 mg/m^2^第1、8天静脉滴注+顺铂25 mg/m^2^第1、2、3天静脉滴注，每21天重复。在治疗过程中发生疾病进展或出现严重毒性反应时停止研究治疗。

### 1.5 疗效评价

疗效按照RECIST标准，分为完全缓解（complete response, CR）、部分缓解（partial response, PR）、疾病稳定（stable disease, SD）及疾病进展（progressive disease, PD）；达到CR或PR者评定为有效，达到CR、PR或SD者评定为疾病控制，毒副反应根据WHO毒性反应评定标准分为0度-4度。

### 1.6 随访与统计学方法

由专人对所有患者进行定期随访，每2周进行电话随访，每4周进行门诊随访，直至患者死亡，随访时间为2007年9月-2010年8月。主要研究终点是总生存时间（overall survival, OS）、一线治疗的无疾病进展生存期（progression-free survival, PFS）和安全性。OS从诊断明确开始到患者死亡，PFS从接受化疗开始至疾病进展。所有统计分析均使用SPSS 17.0统计软件完成，率和构成比的组间比较采用*χ*^2^检验，采用*Kaplan-Meier*法统计生存期和无疾病进展生存期，并采用*Log-rank*检验比较组间差异，*P* < 0.05为差异有统计学意义。

## 2 结果

### 2.1 治疗完成情况

随访至2010年8月，随访时间12周-76周。65例患者至少完成2个周期化疗而进入疗效评价，GP组1例患者因严重皮疹未完成至少2个周期治疗，而GO组无患者因治疗相关不良事件影响后续治疗。GO组33例患者共完成138个周期GO方案治疗，平均完成4.18个周期治疗，72.7%的患者完成至少4个周期治疗；GP组32例患者共完成127个周期GP方案治疗，平均完成3.97个周期治疗，60.6%的患者完成至少4个周期治疗。GO组患者完成治疗情况优于GP组患者，且总体耐受性更好，生活质量更高。

### 2.2 近期疗效

GO组33例中CR 0例，PR 12例，SD 12例，PD 9例，疾病控制率为72.7%（24/33）。GP组32例中CR 0例，PR 13例，SD 8例，PD 11例，疾病控制率为65.6%（21/32）。经过统计检验，GO组与GP组在近期有效率（36.4% *vs* 40.6%, *P*=0.801）和疾病控制率（72.7% *vs* 65.6%, *P*=0.598）方面的差异无统计学意义（表 2）。

**2:** 本研究纳入患者疗效评定（*n*=65） Efficacy evaluation of patients included in this study (*n*=65)

Efficacy	GO group (*n* =33)	GP group (*n* =32)	*P*
CR	0	0	
PR	12	13	
SD	12	8	
PD	9	11	
Efficacy	12 (36.4%)	13 (40.6%)	0.801
Clinical stability	24 (72.7%)	21 (65.6%)	0.598
PFS (week)	24	18	0.565
OS (week)	44	36	0.918
CR: complete response; PR: partial response; SD: stable disease; PD: progressive disease; PFS: progression-free survival; OS: overall survival.			

### 2.3 生存情况

GO组病例的中位PFS为24周（95%CI: 21.051-26.949），中位OS为44周（95%CI: 37.322-50.678）；GP组病例的中位PFS为18周（95%CI: 8.496-27.504），中位OS为36周（95%CI: 30.456-41.544）。经过统计检验，两组间的中位PFS（24周*vs* 18周，*P*=0.565，图 1A）和中位OS（44周*vs* 36周，*P*=0.918，图 1B）的差异无统计学意义。

**1:**
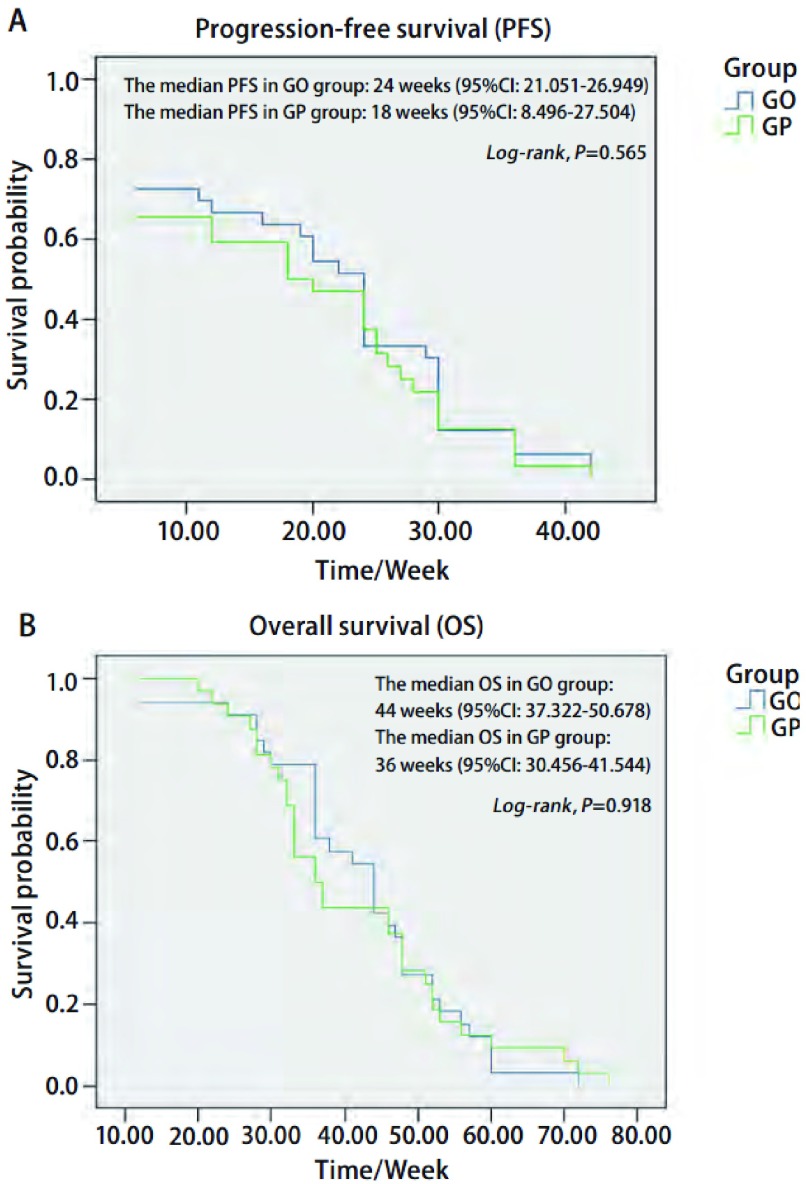
GO组和GP组的中位无疾病进展期（A）和中位生存期（B）的比较 The comparison of progression-free survival (PFS) (A) and overall survival (OS) (B) in GO and GP groups

### 2.4 毒性反应

全部66例患者均可评价毒性反应。两组具有类似的主要毒性反应，包括骨髓抑制、胃肠道反应和神经毒性等，但多数为1级-2级毒性反应。值得注意的是，GO组较GP组更易发生1级-2级神经毒性（78.8% *vs* 9.1%, *P* < 0.001），但通常为轻度且完全可逆，处理后均不影响继续治疗。与安全性密切相关的3级-4级毒性反应发生情况见表 3。GP组3级-4级贫血（33.3% *vs* 0, *P* < 0.001）和恶心呕吐（27.3% *vs* 0, *P*=0.004）发生率均明显高于GO组。本研究中两组患者均未出现治疗相关性死亡。

**3:** Ⅲ-Ⅳ级毒性反应（*n*=66） Toxicities of grade Ⅲ-Ⅳ (*n*=66)

Toxicity	GO group (*n* =33)	GP group (*n* =33)	*P*
Leukopenia	2 (6.1%)	3 (9.1%)	> 0.999
Thrombocytopenia	5 (15.2%)	4 (12.1%)	> 0.999
Anemia	0	11 (33.3%)	< 0.001
Nausea/vomiting	0	9 (27.3%)	0.004
Impaired liver function	0	0	
Impaired renal function	0	0	
Neurotoxicity	0	0	
Rash	0	1 (3.0%)	> 0.999

## 3 讨论

以顺铂为代表的铂类药物是晚期NSCLC化疗的基本药物，但其较强的毒副作用成为老年患者应用的障碍。2003年ASCO肺癌指南推荐老年晚期NSCLC患者接受第三代细胞毒性药物单药化疗；但近年来研究^[1, 2]^发现，老年患者可以耐受更积极的化疗方案且获益更大。奥沙利铂是继顺铂、卡铂（carboplatin, CBP）之后的广谱第三代铂类抗肿瘤药，可与DNA形成铂链加合物，从而阻断DNA的复制和转录，抑制肿瘤细胞增殖^[3]^。奥沙利铂单药对NSCLC也有客观的疗效^[4]^。更有研究^[3, 5]^证实：奥沙利铂对顺铂、卡铂耐药的多种细胞株系同样表现出有效的抗瘤作用，尤其是对顺铂耐药的细胞株有明显的抑制作用。此外，奥沙利铂使用方便，无需水化，具有更好的耐受性。因此奥沙利铂为老年晚期NSCLC患者采用铂类双药化疗提供了新的治疗选择。

本研究中吉西他滨联合奥沙利铂或顺铂一线治疗老年晚期NSCLC有效率分别为36.4%和40.6%，略高于国外既往文献^[6, 7]^报道，可能与本研究65例患者均为初治、无明显耐药问题且样本量较小有关。Grossi等^[8]^报道：肿瘤控制率较近期有效率而言与中位OS有更好的相关性。Chevalier^[9]^认为中位OS的少量增加，即便是1个月-2个月，亦可以代表 20%-30%患者OS增加6个月。本研究中GO组肿瘤控制率为72.7%，中位PFS为24周，中位OS为44周；GP组肿瘤控制率为65.6%，中位PFS为18周，中位OS为36周。GO方案较GP方案在肿瘤控制率、中位PFS、中位OS上显示了一致的临床获益倾向。本研究结果支持大规模多中心的研究作进一步验证。

奥沙利铂单药运用具有较高的安全性和耐受性，且与许多化疗药物联合用药时毒性不叠加^[10]^。奥沙利铂的神经毒性主要包括急性的和剂量累积性的。急性的神经毒性通常无需处理即可恢复^[11, 12]^。累积性的神经毒性与剂量有关，一般停药后会逐渐恢复^[11]^。本研究中GP组3级-4级贫血（33.3% *vs* 0, *P* < 0.001）和恶心呕吐（27.3% *vs* 0, *P*=0.004）发生率均明显高于GO组。虽然GO组1级-2级神经毒性发生率明显高于GP组（78.8% *vs* 9.1%），但GO组患者完成计划治疗情况优于GP组（72.7% *vs* 60.6%），提示患者对GO方案总体耐受更好，不良反应更轻微，从化疗中获益可能性更大。目前，老年晚期NSCLC患者的最优化疗方案还需要进一步验证。

2010年ASCO年会评出的临床肿瘤学年度重大进展之一就是关于老年晚期NSCLC的一线化疗。该进展源自IFCT-0501研究^[13]^，首次证实了对于PS评分为0分-2分的老年晚期NSCLC患者，卡铂联合紫杉醇化疗的疗效优于以往的单药治疗方案，为老年晚期NSCLC的一线治疗提供了新的循证证据。2005年发表的一项研究^[14]^显示，对于老年晚期NSCLC，吉西他滨联合奥沙利铂方案有较好的近期疗效，毒副作用轻，治疗耐受性好。但该研究38%的患者为复治，并不是针对NSCLC的一线化疗。此外，虽然比较了GO、GP两种方案的OS，但并未比较PFS。本研究的患者全部为70岁以上老年晚期NSCLC患者，PS评分为0分-2分，入组前未接受其他抗肿瘤治疗，避免了一些混杂因素的干扰。由于OS容易受到后续治疗混杂因素的干扰，故亦将PFS作为重要观察指标。因此，本研究正是对上述研究的一种必要补充。

## 4 结论

吉西他滨联合奥沙利铂与吉西他滨联合顺铂治疗老年晚期NSCLC相比较有相似的疗效；而毒性反应低于后者，临床应用更安全，是一线治疗老年晚期NSCLC的较好方案，值得进一步研究。
